# Molecular changes to tendons after collagenase-induced acute tendon injury in a senescence-accelerated mouse model

**DOI:** 10.1186/s12891-019-2488-1

**Published:** 2019-03-21

**Authors:** Yasuhiro Ueda, Atsuyuki Inui, Yutaka Mifune, Fumiaki Takase, Takeshi Kataoka, Takashi Kurosawa, Kohei Yamaura, Takeshi Kokubu, Ryosuke Kuroda

**Affiliations:** Department of Orthopaedic Surgery, Kobe University Graduate School of Medicine, 7-5-1 Kusunoki-cho, chuo-ku, Kobe, 650-0017 Japan

**Keywords:** Aging, Acute tendon injury, Tendon degeneration

## Abstract

**Background:**

Aging impairs tendon healing and is a potential risk factor for chronic tendinitis. During normal aging, tendons undergo structural and biomechanical degenerative changes, accompanied by a reduction in the number of tenocytes and changes to their properties. However, molecular changes in aged tendons under inflammatory conditions are not well understood. The present study analyzed the molecular changes in collagenase induced acute tendon injury using a senescence-accelerated mouse (SAM) model.

**Methods:**

SAMP6 mice were used as an aging animal model and SAMR1 mice were used as a control to represent a senescence-resistant inbred strain. All the mice used in the study were 40 weeks old. Collagenase I from *Clostridium histolyticum* (20 μL) was injected percutaneously to the tendon-bone junction of the Achilles tendon. Two weeks after treatment, the Achilles tendons were harvested and stained using Picrosirius Red to determine collagen expression. Real-time PCR was performed to analyze gene expression of IL-6, tenomodulin, type I and type II collagen, MMP-9, TIMP-1, and TIMP-2.

**Results:**

Collagenase injection resulted in significantly higher gene expression of IL-6 but significantly lower tenomodulin expression compared with the control in SAMP6 and SAMR1 mice. In SAMP6 mice, gene expression of type III collagen and MMP-9 was significantly higher in the collagenase-injected group compared with the control group. SAMP6 mice also showed lower expression of type I collagen, TIMP-1, and TIMP-2 in the collagenase-injected group compared with the control group. Picrosirius Red staining showed the highest expression of type III collagen in the collagenase-injected SAMP6 group compared with the other groups.

**Conclusions:**

The collagenase-injected SAMP6 group showed higher expression of IL-6, MMP-9, and type III collagen and lower expression of type I collagen, TIMP-1, and TIMP-2, which are known to suppress metalloproteinases. The results indicate that aging may lead to dysfunction of the tendon healing process after acute tendon injury.

## Background

Aging is associated with impaired tendon healing and is a potential risk factor for chronic tendinitis, as suggested by the high prevalence of rotator cuff disorders in the elderly [1]. Aging has also been shown to be an important factor associated with the probability of re-tear [2]. During normal aging, tendons undergo structural and biomechanical degenerative changes, accompanied by a reduction in the number of tenocytes, as well as changes to their properties [3]. A recent increase in health awareness has seen an increase in the number of elderly people participating in sports; however, there is a potential risk of tendon injury or overuse, such as tendinitis, in this population. Although, there have been several reports on the molecular changes to tendons during normal aging, molecular changes in aged tendons under inflammatory conditions are not well understood. Matrix metalloproteinases (MMPs) and their inhibition by tissue inhibitor of metalloproteinases (TIMPs) influence the degree of degradation as well as the production and remodeling of the tendon extracellular matrix [4]. In aged tendon, compared with those of younger people, the normal level of MMP-2 and MMP − 9 are higher [5] and mechanical stress cause higher MMP activity [6]. Diminished collagen content and mRNA expression of type I and type III collagen have been reported in aged rat Achilles tendons [5]. To date, there have been no reports regarding MMP activity and collagen synthesis under inflammatory conditions in the aged tendon. In the present study, we used a senescence-accelerated mouse (SAM) model to analyze the molecular changes in collagenase induced acute tendon injury.

## Methods

### Animal models

All the animal procedures were performed under the approval and guidance of the Animal Care and Use Committee at our institution.As an aging animal model, we used senescence-accelerated mouse prone (SAMP) mice, which are a widely used aging muscle model. The SAM model has been developed at Kyoto University since 1970 through the selective inbreeding of the AKR/J strain of mice, donated by the Jackson Laboratory in 1968, based on a graded score for senescence, life span, and pathologic phenotype [7]. The SAM strain has a short life span and strain-specific pathological features and is an established experimental animal model for age-associated disease research in systematically designed studies [8]. As a control, we used SAMR (senescence-accelerated mouse resistant) mice to represent a senescence-resistant inbred strain. Among several SAM subtypes, SAMP6 is used as a murine model of senile osteoporosis as it has low bone mass in the vertebra, tibia, and femur [9]. Since the strain has been widely used in orthopedic research, the SAMP6 strain was used as an aged acute tendon injury model. In a 32 weeks old SAMP6 mouse, a reduction in the number of fibroblasts and the diameter of the tendon collagen fibers was reported [10]. We used 40-week-old SAMP6 and SAMR1 male mice (*n* = 24 for each strain). The mean body weight of the SAMR1 and SAMP6 mice was 36.6 ± 1.86 (34.2–39.9) g and 45.7 ± 4.53 (39.5–53.6) g, respectively. Male mice were used in this study since previous studies indicated estrogen levels in female mice play a crucial role in tendon metabolism and alter the production of various growth factors [11]. SAMP6 and SAMR1 mice were purchased from Japan SLC (Shizuoka, Japan). They were housed in standard laboratory conditions (12-h light:12-h dark cycle, room air temperature 22–24 °C, two mice per cage) and had free access to tap water and food pellets.

### Acute tendon injury model

Twenty four SAMR1 mice and twenty four SAMP6 mice were used in the study. After anesthesia using isoflurane (Wako, Osaka, Japan), 0.3 mg of collagenase I from *Clostridium histolyticum* (Sigma Aldrich, Ca., USA) was dissolved in 20 μL of phosphate buffered saline and injected into the mid-portion of the left Achilles tendon of all mice using a 30-gauge needle [12, 13]. The contralateral limb was injected 20 μL of phosphate buffered saline and used as a control. Free cage activity was allowed after injection. All the animals survived until they were sacrificed. At two weeks after injection, all mice were euthanized with an overdose of isoflurane (Wako, Osaka, Japan) and intraperitoneal injection of pentobarbital sodium (Kyoritsu Seiyaku, Tokyo, Japan). The Achilles tendon was harvested to analyze gene expression and histological evaluation.

### Quantitative real-time polymerase chain reaction (PCR)

The Achilles tendons were carefully isolated from contaminating connective tissue and minced. Total RNA was extracted from the tendons using an RNeasy Mini Kit (Qiagen, Valencia, CA, USA) with homogenizer then reverse transcribed to produce single-stranded cDNA using a high-capacity cDNA reverse transcription kit (Applied Biosystems, Foster City, CA, USA). Real-time PCR was performed in duplicate with cDNA samples using a fast real-time PCR system and SYBR Green reagents to analyze mRNA expression of interleukin-6 (IL-6), tenomodulin, type III collagen, MMP-9, TIMP-1, and TIMP − 2. The results were normalized to mRNA levels of the housekeeping gene glyceraldehyde 3-phosphate dehydrogenase (GAPDH) and were expressed relative to their levels in normal culture levels using the 2^(−∆∆ CT)^ method (*n* = 16 in each group).

The oligonucleotide primer sequences used for real-time PCR amplification were as follows:

IL-6, 5′- TAGTCCTTCCTACCCCAATTTCC -3′ (forward) and 5′- TTGGTCCTTAGCCACTCCTTC -3′ (reverse); tenomodulin, 5′- TGTACTGGATCAATCCCACTCT -3′ (forward) and 5′-GCTCATTCTGGTCAATCCCCT -3′ (reverse); type I collagen, 5-CCA GCG AAG AAC TCA TAC AGC-3′ (forward) and 5′- GGA CAC CCC TTC TAC GTT GT-3′ (reverse); type III collagen, 5′- TGG AGA CAG GTC AGA CCT G-3′ (forward) and 5′- TAT TCG ATG ACT GTC TTG CC-3′ (reverse); MMP-9, 5′- TGAATCAGCTGGCTTTTGTG -3′ (forward) and 5′- ACCTTCCAGTAGGGGCAACT -3′ (reverse); TIMP-1, 5′- GCAACTCGGACCTGGTCATAA -3′ (forward) and 5′- CGGCCCGTGATGAGAAACT -3′ (reverse); TIMP-2, 5′- TCAGAGCCAAAGCAGTGAGC -3′ (forward) and 5′- GCCGTGTAGATAAACTCGATGTC -3′ (reverse); and GAPDH, 5′- GGT GGT CTC CTC TGA CTT CAA CA-3′ (forward) and 5′- GTT GCT GTA GCC AAA TTC GTT GT-3′ (reverse).

### Picrosirius red staining for collagen expression

Picrosirius Red staining was performed to analyze the orientation of collagen fibers in the tendons. Frozen, long-axis sections of Achilles tendons embedded in Optimal Cutting Temperature (OCT) compound (Sakura Finetek USA, Inc., Torrance, CA, USA) were serially cut into 7-μm-thick sections and fixed using 10% phosphate-buffered paraformaldehyde at room temperature for 15 min. Tissues processed for histology were stained using a Picrosirius Red staining kit (Polyscience, Inc. Warrington, PA) according to the manufacturer’s instructions. A light microscope fitted with a light polarizer was used to capture the digital images. The organization of the Achilles tendon collagen fibers was visualized by turning the polarizer to maximize the signal in the Achilles tendon. Picrosirius Red stains type I collagen yellow and type III collagen green. For semiquantitative evaluation of collagen expression, we measured the chromaticity of yellow for type I collagen (*n* = 8 in each group).

### Statistical analysis

The data is presented as mean ± standard deviation (SD) of independent experiments. The Excel statistical software package (Ekuseru-Toukei 2015; Social Survey Research Information Co., Ltd., Tokyo, Japan) was used to perform all the statistical analyses of the recorded data. The data were expressed as the n-fold difference from the SAMR1 group. For a comparison of the two groups, two-way analysis of variance (ANOVA) was performed. Post hoc analysis was performed by Fisher’s protected least significant difference test. A value of *p* < 0.05 was considered significant.

## Results

**Fig. 1:**
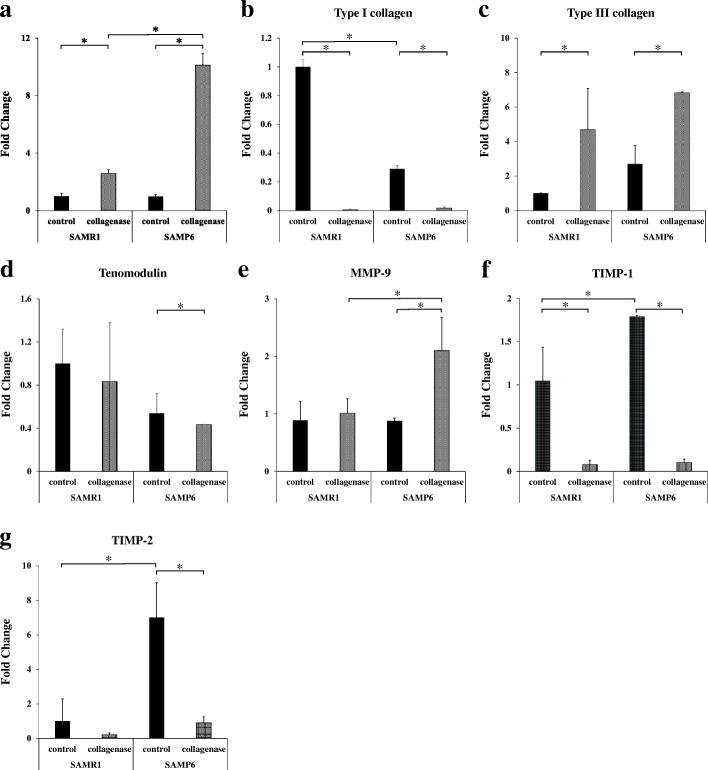
mRNA expressions in the Achilles tendons of SAMR1 and SAMP6. **a** IL-6 is a marker for inflammation, **b**, **c** type I and type III collagen are markers for degeneration, and **d**, **e**, **f**, **g** tenomodulin, MMP-9, TIMP-1, and TIMP-2 are markers for tendon remodeling. (*p < 0.05). **a**. mRNA expression of IL-6. **b**. mRNA expression of type I collagen. **c**. mRNA expression of type III collagen. **d**. mRNA expression of tenomodulin. **e**. mRNA expression of MMP-9. **f**. mRNA expression of TIMP-1. **g**. mRNA expression of TIMP-2

In both SAMP6 and SAMR1 mice, collagenase injection triggered tendon inflammation resulting in increased expression of IL-6 and MMP-9 compared with the contralateral (control) limb. Expression of IL-6 and MMP-9 was significantly higher in the SAMP6 mice compared with the controls (Fig. 1a and e). In the control groups, expression of type I collagen (a tendon matrix marker) was significantly lower in the SAMP6 mice compared with the SAMR1 mice. Type I collagen expression in the collagenase-injected group was significantly lower than the control group both in SAMR1 and SAMP6 mice (Fig. 1b). Type III collagen was increased in both SAMP6 and SAMR1 mice after collagenase injection (Fig. 1c). On the other hand, tenomodulin expression was decreased after collagenase injection in the SAMP6 group, although a statistically significant decrease was not seen in the SAMR1 group (Fig. 1d). In control tendons, expression of TIMP-1 and TIMP-2 was statistically higher in SAMP6 mice compared with SAMR1 mice. Collagenase injection decreased TIMP-1 and TIMP-2 expression in both SAMR1 and the SAMP6 mice (Fig. 1f and g). There was no significant difference in TIMP-1 and TIMP-2 expression between the collagenase-injected SAMR1 and the SAMP6 mice. The measured value are shown in Table 1.

**Table 1:** mRNA expressions in the Achilles tendons of SAMR1 and SAMP6

(a) IL-6				
	SAMR1		SAMP6	
	control	collagenase	control	collagenase
value	1.00	2.59	0.99	10.12
SD	0.21	0.25	0.13	0.79
(b) Type I collagen				
	SAMR1		SAMP6	
	control	collagenase	control	collagenase
value	1.00	0.01	0.29	0.02
SD	0.05	0.00	0.02	0.01
(c) Type III collagen				
	SAMR1		SAMP6	
	control	collagenase	control	collagenase
value	1.00	9.35	1.19	6.11
SD	0.02	1.63	1.03	1.25
(d) Tenomodulin				
	SAMR1		SAMP6	
	control	collagenase	control	collagenase
value	1.00	0.83	0.54	0.43
SD	0.32	0.55	0.16	0.00
(e) MMP-9				
	SAMR1		SAMP6	
	control	collagenase	control	collagenase
value	1.00	1.14	0.99	2.37
SD	0.32	0.25	0.05	0.58
(f) TIMP-1				
	SAMR1		SAMP6	
	control	collagenase	control	collagenase
value	1.00	0.07	1.31	0.06
SD	0.15	0.04	0.01	0.02
(g) TIMP-2				
	SAMR1		SAMP6	
	control	collagenase	control	collagenase
value	1.00	0.21	7.01	0.90
SD	1.29	0.09	2.01	0.35

**Table 2:** Expression of type I collagen was semiquantitatively analyzed by the chromaticity of yellow in Fig. 2

	SAMR1		SAMP6	
	control	collagenase	control	collagenase
value	1.00	0.49	0.43	0.03
SD	0.29	0.15	0.10	0.02

**Fig. 2:**
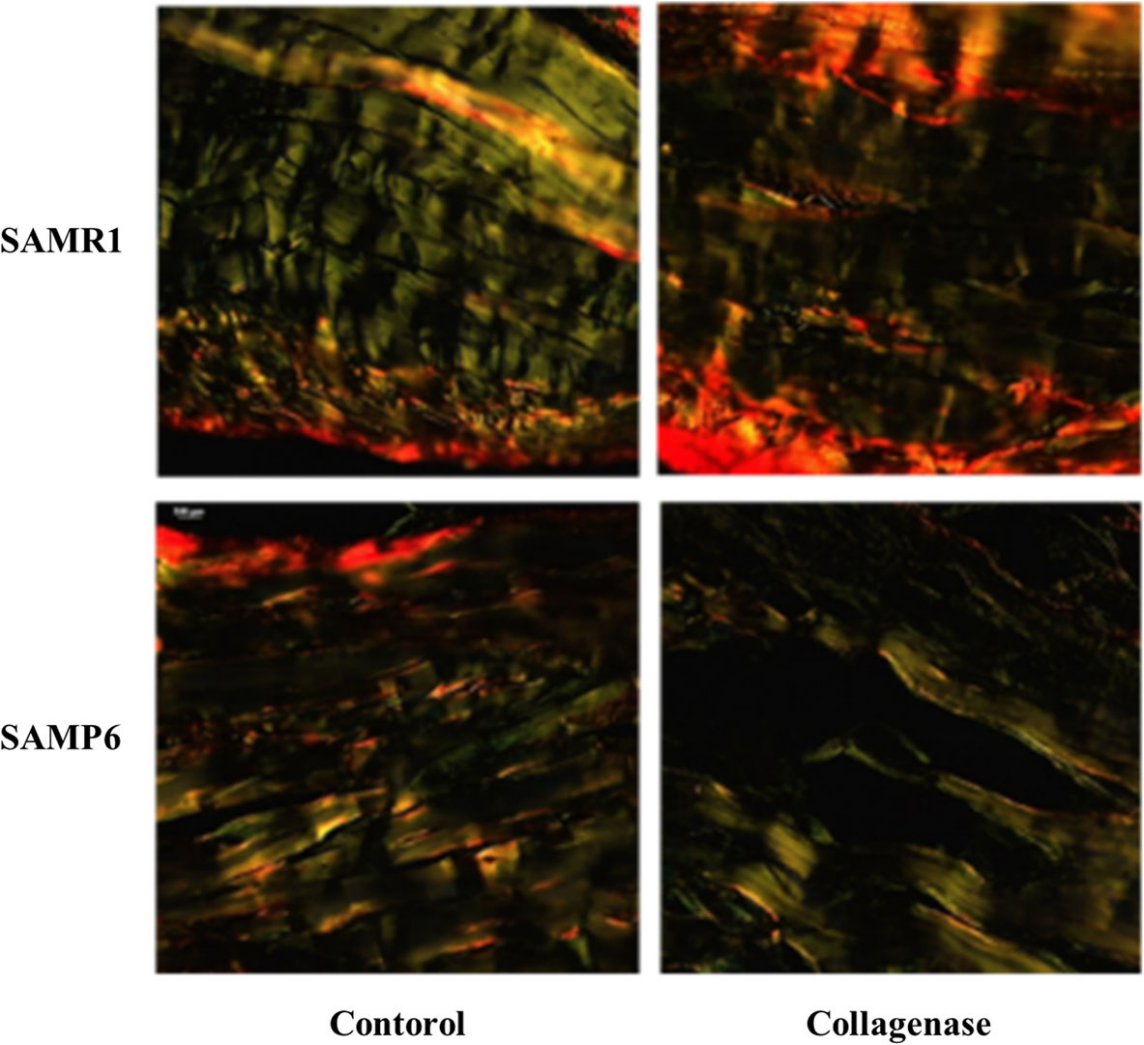
Collagen arrangement in the Achilles tendon stained using Picrosirius Red. Yellow represent type I collagen and green represents type III collagen

**Fig. 3:**
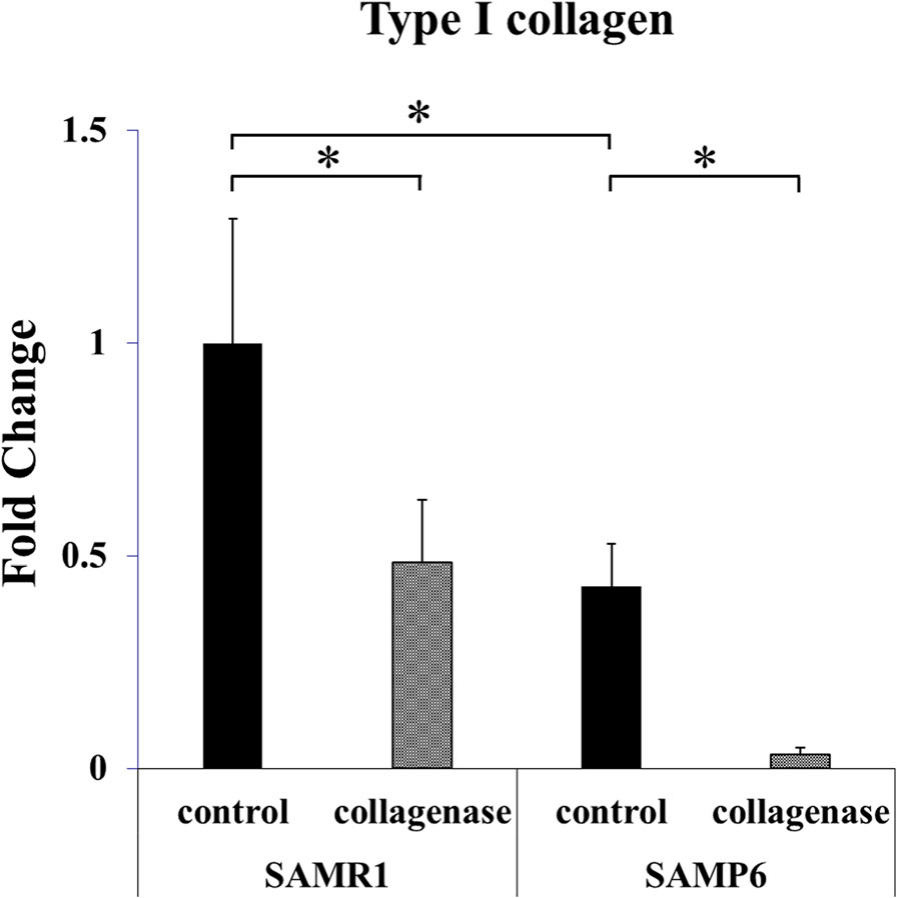
Expression of type I collagen in the Achilles tendon detected by Picrosirius Red staining. Expression of type I collagen was semiquantitatively analyzed by the chromaticity of yellow in Fig. 2. (*p < 0.05)

Picrosirius Red staining showed strong expression of type I collagen in the SAMR1 control group. On the other hand, expression of type I collagen was decreased in SAMP6 mice, especially after collagenase injection (Fig. 2). Quantitative analysis showed that type I collagen expression decreased following collagenase injection especially in SAMP6 mice (Fig. 3, Table 2).

## Discussion

Tendinitis is defined as the inflammation of a tendon and is the first stage of tendinosis, which is a degeneration of the tendon’s collagen in response to chronic overuse or aging. IL-6 is an important reactive cytokine expressed during inflammation [14]. Expression of some IL-6 family members is upregulated in the pathological tendon [15]. The physiological role of IL-6 has mostly been studied in the context of the acute phase response, although there is growing evidence that IL-6 also plays a central role in the pathogenesis of chronic disease [16]. Collagen metabolism has also been shown to be influenced by IL-6 [17]. In the present study, IL-6 was analyzed to evaluate the degree of collagenase-induced inflammation in tendons. There is no significant difference in IL-6 mRNA expression between SAMR1 and SAMP6 in control groups (SAMR1, 1.0 ± 0.21; SAMP6, 0.99 ± 0.13). These results resemble the previous report that there is no difference between the young rats and aged rats in the serum IL-6 level [18]. Therefore, the baseline levels of tendon inflammation in these aging mice (SAMP6) and control mice (SAMR1) have no difference. Gene and protein expression of IL-6 increased in acute human RC and Achilles tendon tear [15, 19, 20], which continued until two weeks post-surgical repair [21]. On the other hand, its expression after acute tendon injury in the aged mouse is not known. In the present study, both SAMR1 and SAMP6 mouse showed upregulation of IL-6 after collagenase injection.

Compared with collagenase-injected SAMR1 mice, there was a strong upregulation of IL-6 in SAMP6 mice. This shows that more severe inflammation was induced by collagenase injection in the Achilles tendons of the aged mouse group.

Tenomodulin is a mature marker for tendon and ligament lineage cells. Its absence leads to an inferior tendon repair process, resulting in adipocyte accumulation and fibrovascular scar formation during early tendon healing [22]. The ability of tendon-derived stem/progenitor cells to differentiate into tenocytes also diminishes with age [23, 24]. Expression of tenomodulin was lower in aged TSPCs compared with young cells [23]. But, there are few previous reports that compare tenomodulin expression of aged with young in early phase from tendon injury.

In the present study, tenomodulin expression was lower in SAMP6 mice and was decreased by collagenase injection. This result indicates that tendon repair ability is decreased in aged tendons under inflammatory condition compared with younger tendons.

Matrix turnover involves the synthesis and degradation of matrix components and is important for the maintenance and repair of tendons. Decreased collagen turnover and altered gene expression of the extracellular matrix was reported in aged tendon [25]. In normal tendons, approximately 90% of the collagen is type I, whereas type III collagen is upregulated during inflammation [26]. A study using human Achilles tendon showed that levels of type III collagen mRNA were significantly higher in tendons with chronic pain or spontaneous rupture compared with normal tendons [27]. Approximately 90% of the collagen in normal tendons is type I. In tendinopathic and ruptured Achilles tendons; there is a reduction in the proportion of type I collagen, and a significant increase in the amount of type III collagen. [28] Microtrauma within the tendon may heal by the production of type III collagen, which is an abnormal healing response. [28] In the present study, the expression of type III collagen in collagenase injected groups is lower in SAMP6 than SAMR1. This may indicate that SAMP6 has lower potential to express type III collagen during its healing process after tendon injury.

MMPs play a key role in regulating matrix remodeling and are considered responsible for the degradation of collagens and proteoglycans [29, 30]. The present study revealed that the expression of MMP-9 was higher in the tendons of aged mice compared with those of younger mice. A similar age-dependent increase in MMP-2 or MMP-9 activity was also reported in skin, heart, articular cartilage, and even plasma [31–34].

TIMPs are tissue-specific, endogenous inhibitors of MMPs. All known MMPs are inhibited by all TIMPs; however, the efficacy of MMP inhibition varies [35]. For example, TIMP-1 strongly inhibits many MMPs except for membrane-type MMPs, including MMP-14, 15, 16, 19, and 24 [35]. There is considerable evidence supporting an inhibitory effect of TIMP-2 on extracellular matrix proteolysis in several tissues [36, 37]. Our study showed that the expressions of TIMP-1 and TIMP-2 are increased in SAMP6 tendons compared to SAMR1 in control group. Collagenase injection decreased these expressions both in SAMR1 and SAMP6. The balance of MMPs and TIMPs is important for tendon matrix turnover. In the present study, an imbalance of these tendon remodeling factors was observed in the aged inflammatory tendon. We believe that under the inflammatory conditions of the aged tendon, the imbalance of MMPs and TIMPs negatively affected tendon matrix turnover, leading to altered expression of type I and type III collagen, representing a weaker remodeling ability.

There are several limitations to this study. The SAMP6 mouse model is not a normal aging model. Although it is not a transgenic mouse, some strains show amyloidosis, immunodeficiency, renal atrophy, and learning disabilities. SAMP6 mice spontaneously develop osteoporosis or sarcopenia early in life and are used in musculoskeletal research. In the future, a similar study using a normal strain could confirm the present results. Biomechanical evaluation of the Achilles tendon of the aged mouse was not performed in the present study due to limited animal numbers. Further studies are required to determine the relevance of aging on the mechanical properties of the tendon.

## Conclusion

The aged tendon showed increased inflammation and decreased tendon remodeling. Therefore, aging may have a suppressive effect on the tendon healing process. The results indicate that aging may lead to dysfunction of the tendon healing process after acute tendon injury.

## Abbreviations

cDNA: Complementary deoxyribonucleic acid
GAPDH: Glyceraldehyde 3-phosphate dehydrogenase
IL-6: Interleukin-6
MMP: Matrix metalloproteinase
OCT: Optimal cutting temperature
PCR: Polymerase chain reaction
RNA: Ribonucleic acid
SAM: Senescence-accelerated mouse
SAMP: Senescence-accelerated mouse prone
SAMR: Senescence-accelerated mouse resistant
SD: Standard deviation
TIMP: Tissue inhibitors of metalloproteinase
TSPC: Tendon stem/progenitor cell
